# Latent Transition Analysis of Motor Development Patterns in Preschoolers

**DOI:** 10.3390/children10050777

**Published:** 2023-04-25

**Authors:** Hsueh-Chin Chao, Chun-Ta Lin, Jui-Hung Tu, Chung-Chin Wu

**Affiliations:** 1 Physical Education Office, National Kaohsiung University of Science and Technology, Kaohsiung 80778, Taiwan; 2 Office Physical Activities, National Pingtung University, Pingtung City 900391, Taiwan; st8871210913@gmail.com; 3 Department of Physical Education, National Pingtung University, Pingtung City 900391, Taiwan; 4 Department of Early Childhood Education, National Pingtung University, Pingtung City 900391, Taiwan

**Keywords:** latent profile analysis, latent transition analysis, motor development, pattern analysis, preschooler

## Abstract

In-group heterogeneity is often neglected during investigations of motor development patterns in children. Moreover, the variation in motor development patterns over time has seldom been examined. In this work, 1884 three-year-old preschoolers were selected from a panel study conducted in Taiwan called the National Longitudinal Study of Child Development and Care. A confirmatory factor analysis was applied to analyze the construct validity of the assessments of motor development used for these children. A latent profile analysis and latent transition analysis (LTA) were sequentially applied to clarify their motor development patterns at the ages of three and four years and their transitions between these two ages. The following findings were obtained: (1) The motor development assessment had good validity. (2) Considerable heterogeneity regarding motor development in preschoolers was observed, in which four and three subgroups displaying distinct levels of mastery with respect to their gross and fine motor skills were identified at the ages of three and four years, respectively. (3) From age three to age four, a large proportion of the preschoolers exhibited improvements or retentions in both gross and fine motor skills, whereas some of the preschoolers were classified into subgroups displaying “gross motor retention and fine motor progression,” “gross motor progression and fine motor retention,” “gross motor retention and fine motor regression,” and “gross motor regression and fine motor progression.” Few preschoolers exhibited “general motor regression.” The present results suggest that there were considerable heterogeneous groups in the motor development in preschoolers in the middle of early childhood, and this phenomenon has rarely been addressed in former studies. The LTA results implied that effective interventions should be given sequentially to preschoolers in subgroups whose motor development presented regression and retention tendencies.

## 1. Introduction

Motor development in preschoolers is important for their daily learning engagement, physical activities, and cognitive development. For example, gross motor development in early childhood has been reported to be related to later cognitive performance [1]. Therefore, it is crucial for researchers and practitioners to understand the current status of motor development in preschoolers to plan interventions and examine their effects. In a previous work, Blauw-Hospers and his colleagues systematically reviewed 34 empirical studies to investigate the effects of intervention programs on motor development in young children with ages from zero to three years old [2]. The results revealed that approximately 60% of the studies (21 out of 34) found no significant effects of intervention on the children’s motor development. This may suggest that one intervention cannot fit all children with varying levels of motor development. In other words, it may imply that children are not a homogeneous group with respect to motor development, and the positive effects of intervention observed in a certain group may be offset by negative effects in other group(s) when they are taken together as a homogeneous group in an analysis. However, most previous studies investigating motor development in children used statistical techniques that assume homogeneity (e.g., analysis of variance, ANOVA) and ignore group heterogeneity, which may have led to misleading results [3,4,5,6,7,8,9].

In these former studies, Nahar and his colleagues introduced z-tests that assumed group homogeneity to examine group differences (e.g., stunted vs. non-stunted, underweight vs. non-underweight) in motor development in infants and young children at 6, 15, and 24 months [4]. Kokštejn and his colleagues assessed gender differences in fundamental motor skills proficiency through a Mann–Whitney U test [5]. Similarly, in another study, in addition to gender differences in motor coordination, the differences in this skillset were compared among three age groups (6–7, 8–10, and 11–13 years) and among four different weight statuses (underweight, normal weight, overweight, and obese) [6]. With a slight difference from other studies in regard to the causes of differences in motor development, the effects of two movement skill training programs on the movement skills of 10- to 11-year-old children were compared through the implementation of an analysis of covariance (ANCOVA) [7]. In addition, a predictive study used a regression analysis to examine the effects of the level of motor development in children at the lower elementary school level on their motor skills at 12 years [8]. In summary, most studies regarding motor development have primarily involved samples below age 3 or above age 5, and these studies mostly considered mean level differences between/among known groups (e.g., gender groups) or between/among groups divided by arbitrary value(s) (e.g., underweight, normal weight, overweight). Therefore, latent heterogeneity in motor development in these samples was not identified, and this may result in inaccurate results and conclusions.

In practice, various aspects of motor development in children may co-occur simultaneously. For example, children may develop fine motor skills to use scissors to cut paper, and they may also develop gross motor skills to coordinate their bodies to engage in physical activities. Similarly, children may also use sequential movements involving both fine and gross motor skills to master tasks [9]. For instance, children have to use their fingers to grasp a ball (fine motor skills) and then swing their arm to throw the ball (gross motor skills) to complete the whole throwing movement. Therefore, investigations of motor development should simultaneously consider different aspects. However, most empirical studies have investigated motor development in children by dividing it into several aspects and treating these aspects as variables [1,2,9]. For example, one study examined the relationships between fundamental motor skills and aspects of academic performance and found that gliding, skipping, and star jumps were correlated with mathematics performance [10]. Few studies have investigated the patterns of motor development and how these patterns change over time for young children. However, as an exception to this generalization, Okuda and his colleagues solely focused on examining the heterogeneity in gross motor development in both ten- and sixteen-year-old children, and identified three latent classes with good, intermediate, and poor balance skills, respectively. They further found that 86% of the children stayed in the good balance motor skill group from 10 to 16 years old, and 75.2% and 62.7% of the members of the intermediate and poor motor skills groups at age 10, respectively, became members of the good motor skills group by age 16 [11].

By adopting mixture analyses (e.g., latent class/profile analyses), several heterogeneous subgroups with different patterns and levels in physical, psychological, and behavioral domains can be identified [12]. For example, several patterns of weight change were observed corresponding to different degrees of weight loss, weight maintenance, and weight gain [13]. In addition, physical activity in older adults was classified into seven subgroups corresponding to different physical activities and activity levels (e.g., “mostly inactive” and “household activities and walking”), and the majority of participants remained in the same subgroups between the waves of the study, indicating similar activity patterns [14]. These findings suggest that subgroups with different patterns and levels of motor development may exist within a preschooler population, and they may present various transitions between the subgroups.

The two purposes of this study are as follows:

(1) To investigate whether there exist subgroups with different patterns and levels of motor development in a preschooler population;
(2) To clarify the transitions between different subgroups of preschoolers with different patterns and levels of motor development.

## 2. Methodology

### 2.1. Participants

First, 2164 preschoolers aged three years (1051 girls, 48.57%) were selected from a panel study conducted in Taiwan called the National Longitudinal Study of Child Development and Care, and this panel study employed the stratified, two-stage probability proportional to size sampling technique. Three hundred and fifty-eight administration districts were integrated into nineteen stratifications and they served as the primary sampling unit, and individuals in these stratifications were in turn to be proportionally selected based on household registration information. Consequently, there were 709, 48, 1140, and 303 parent–preschooler pairs selected, respectively, from middle, Western, Northeastern, and Southeastern Taiwan. A follow-up on the same preschoolers was conducted a year later [12]. After excluding participants missing too many answers on items, there were 1884 preschoolers included in both waves of this study. The preschoolers were registered by their parents.

### 2.2. Motor Development Evaluation Scale

A scale for motor development evaluation developed for preschoolers was used in this study. The scale was composed of two subscales for measuring gross motor development and fine motor development with a total of nine items. The gross motor development subscale comprised two dimensions, namely, “stability and movement” and “body coordination,” which were measured with two and three items, respectively. Representative items for these two dimensions included the following: (1) “he/she can continuously jump forward using both legs” (for stability and movement); and (2) “he/she can throw a ball to hit the target” (for body coordination). There were five items in the gross motor development subscale. The fine motor development subscale also consisted of two dimensions, namely, “grasp and hand operation” and “vision and movement coordination,” which were each measured with two items. Representative items for these two dimensions included the following: (1) “he/she is able to undo a button on a piece of clothing” (for grasp and hand operation) and (2) “he/she is able to draw a straight line” (for vision and movement coordination). There were four items on the fine motor development subscale. Parents were required to choose one of four scores ranging from 1 (“completely unable to do it”) to 4 (“very adept at doing it”) for each item [15,16].

### 2.3. Analysis

Confirmatory factor analysis (CFA), latent profile analysis (LPA), and latent transition analysis (LTA) were implemented using the Mplus 7.4 software to evaluate the construct validity and clarify the motor development patterns and their changes. The maximum likelihood with robust standard errors estimator was used to calculate the parameters.

#### 2.3.1. Confirmatory Factor Analysis

The following indices were used to evaluate the model fit: the chi-square statistic (χ^2^), the comparative fit index (CFI), the Tucker–Lewis index (TLI), and the root-mean-square error of approximation (RMSEA). The insignificant chi-square statistics indicated that the model fit the data well; the chi-square statistics usually reached significant level owing to the large sample size. Therefore, the following criteria were primarily used to evaluate the goodness of model fit: the model was considered to fit the data very well when CFI ≥ 0.95, TLI ≥ 0.95, and RMSEA ≤ 0.06, whereas the model was considered to fit the data only acceptably well when 0.90 ≤ CFI < 0.95, 0.90 ≤ TLI < 0.95, and 0.06 < RMSEA ≤ 0.08 [17,18].

#### 2.3.2. Latent Profile Analysis

LPA was performed prior to LTA to assess heterogeneity in the preschooler population and determine the optimal LPA model for understanding the motor development patterns in the preschoolers at the two time points. Three steps were followed:

(1) Comparison of latent profile models

Three goodness-of-fit indices, namely, the Akaike information criterion (AIC), Bayesian information criterion (BIC), and sample-size-adjusted BIC (ABIC), as well as two likelihood ratio tests, namely, Lo–Mendell–Rubin likelihood ratio test (LMR) and bootstrap likelihood ratio test (BLRT), were used to determine the optimal latent profile model [19]. The model with k latent profiles with the lowest AIC, BIC, and ABIC values and the significant LMR and BLRT test results that compared model with k profile to model with k-1 profile(s) indicated that the goodness of fit of the latent profile model with k classes was significantly improved compared with that of the model with k−1 classes. A simulation study indicated that the BIC and BLRT performed better among these indices [20].

(2) Examination of latent profile classification quality

LPA was used to assign each preschooler to the most likely latent profile according to the conditional probabilities calculated from the responses to the items of the motor development scale. The uncertainty was close to zero if the conditional probability was close to one. A conditional probability of ≥0.70 was considered acceptable. The entropy was also used to evaluate the classification quality, in which an entropy of ≥0.80 was considered good, an entropy between 0.60 and 0.80 was considered acceptable, and an entropy of <0.60 was considered unacceptable.

(3) Naming the latent profiles

After the optimal LPA model had been chosen according to the above criteria, the latent profiles were named based on the motor development patterns observed on the means of the items.

#### 2.3.3. Latent Transition Analysis

At least two waves with identical items are required for conducting LTA, and it is possible that different latent profiles may present owing to the increase in statistical power caused by repeated measures. Therefore, it is necessary to incorporate data from two waves when conducting LTA and to choose the best LTA model according to the results of fitting indices and model comparisons. After the latent profiles in two waves were identified, the transition probabilities were applied to understand the longitudinal changes of the subgroup members in terms of their motor development patterns.

## 3. Results

### 3.1. Construct Validity of the Preschooler Motor Development Scale

The construct validity of the preschooler motor development scale was investigated by the CFA. The results showed that χ^2^(21, *N* = 1884) = 57.01, *p* > 0.05, CFI = 0.99, TLI = 0.99, and RMSEA = 0.030 (with a 90% CI ranging from 0.021 to 0.040). These values indicate that the model fit the data very well, demonstrating good construct validity and the suitability of the scale for evaluating motor development in preschoolers.

### 3.2. Latent Profile Analysis of Motor Development in Preschoolers

The LPA revealed that the model with eight latent profiles displayed the lowest AIC, BIC, and ABIC values (17,407.66, 17,645.93, and 17,509.32, respectively) among the latent profile models of motor development for the three-year-old preschoolers. The results of the likelihood ratio tests also indicated that the goodness of fit for the model with nine latent profiles did not significantly improve compared with that of the model with eight latent profiles (the *p* values for LMR and BLRT were 1, which is larger than 0.05). The entropy for this model was 0.81, which indicated good classification quality. Thus, the model with eight latent profiles appeared optimal for the three-year-old preschoolers. However, after careful inspection of the number and percentage of preschoolers, it was found that one latent profile contained only about 90 preschoolers (less than 5%). There were also three latent profiles with approximately 100 preschoolers. Moreover, the large number of latent profiles was inconsistent with achieving a parsimonious model (i.e., one that explains most of the motor development patterns with few latent profiles). As for the other models, with the exception of the model with one latent profile, all of their entropies were lower than 0.84. The entropies of the models with seven, six, five, and four latent profiles (>0.80) indicated good classification quality. However, the models with six and seven latent profiles each had one latent profile containing few preschoolers (the percentages were 6.74% and 5.73%, respectively), and their entropies were below 0.80. This suggested that the models with six and seven latent profiles were not good models for representing motor development patterns in the preschoolers. Moreover, the entropies of the models with four and five latent profiles were above 0.80 and the proportions of the subgroups were all above 11%. The model with five latent profiles appeared superior to the model with four latent profiles on account of its lower AIC and BIC values and higher entropy (0.83 and 0.82 for the models with five and four latent profiles, respectively). However, the proportions of the subgroups in the model with four latent profiles were considerably greater, suggesting their better suitability for obtaining meaningful results in the subsequent LTA. For the above reasons, the model with four latent profiles was selected as the better model for subsequent analyses.

For the four-year-old preschoolers, the lowest AIC, BIC, and ABIC values were again observed for the model with eight latent profiles (12,277.15, 12,515.42, and 12,378.81, respectively). However, the likelihood ratio tests indicated no significant differences between the models with eight and seven latent profiles (the *p* values for LMR and BLRT were 0.40 and 1, respectively). It appeared that the model with seven latent profiles was optimal, despite the fact that its entropy of 0.89 was considerably lower than those of the models with two, three, and four latent profiles (>0.98). The models with three and four latent profiles each had one subgroup containing few preschoolers (only 2.97% and 0.95%, respectively). By contrast, the proportions of the subgroups in the model with two latent profiles were greater than 20%. However, it seemed that the subgroups were too few in this model to adequately explain motor development in the preschoolers, and the statistical power can be increased to enable more preschoolers to be classified into subgroups in the LTA. Consequently, the model with three latent profiles was temporarily chosen as a basis for the subsequent analysis.

The mean scores for each of the motor development dimensions in the two subscales are presented in Table 1 to allow the naming of the latent profiles. It can be seen that there were four latent profiles for the three-year-old preschoolers, in which the numbers (proportions) of preschoolers in the four latent profiles were 275 (14.60%), 566 (30.04%), 576 (30.57%), and 467 (24.79%). The low, moderate, and high motor development subgroups were defined based on the mean scores ranging from 1 to 2, from 2 to 3, and from 3 to 4, respectively. The first, second, third, and fourth latent profiles were classified as subgroups named “low gross and fine motor development,” “moderate-to-high gross and low fine motor development,” “moderate gross and low-to-moderate fine motor development,” and “high gross and moderate-to-high fine motor development,” respectively.

There were three latent profiles for the four-year-old preschoolers, in which the numbers (proportions) of preschoolers in the three latent profiles were 1494 (79.30%), 334 (17.73%), and 56 (2.97%). According to the mean scores for each of the dimensions, the first, second, and third latent profiles were classified as subgroups named “high gross and moderate-to-high fine motor development,” “moderate gross and fine motor development,” and “low-to-moderate gross and moderate fine motor development,” respectively.

### 3.3. Latent Transition Analysis of Motor Development in Preschoolers

First, a 4-3 latent transition model incorporating the preschoolers at the two time points (t_1_ = 3 years old, t_2_ = 4 years old) was established, in which “4-3” denotes the four and three latent profiles at t_1_ and t_2_, respectively, identified by the LPA. However, it was unclear whether different latent profiles would present at the different time points after incorporating the data from the two waves during the LTA [21]. Thus, it was necessary to clarify this by comparing the goodness of fit between different latent transition models. Therefore, 17 alternative latent transition models were considered in addition to the 4-3 transition model, and the goodness-of-fit indices are listed in Table 2.

As shown in Table 2, the 8-7 transition model displayed the lowest BIC value, which initially suggested that this may be the optimal theoretical model for further examining the latent transitions of motor development. However, further inspection of the various transition conditions revealed that several of the cells contained zeros, indicating that no preschoolers moved from the certain profile in the first wave to the particular profile in the second wave. Moreover, the values of some transition probabilities in the cells were extremely small (e.g., 0.01). There were even 26 and 42 (out of 56) cells with transition probabilities of less than 0.01 (46.43%) and 0.03 (75%), respectively. The other transition models afforded similar or even worse results. For example, seven of the sixteen cells in the 4-4 transition model exhibited transition probabilities of less than 0.03 (43.75%).

There were three out of twelve cells in the 4-3 transition model, one out of six cells in the 3-3 transition model, and two out of six cells in the 2-3 transition model with transition probabilities of less than 0.03. The 3-2 transition model had the fewest cells with a transition probability of less than 0.03, and only the probabilities of correct classification in this model were lower than 0.80. By contrast, the probabilities of correct classification in the 2-3, 3-3, and 4-3 transition models were above 0.80, and the probabilities in the 2-3 and 4-3 transition models were 0.853 and 0.861, respectively. The 4-3 transition model was selected as the optimal model for understanding the latent transitions of motor development in preschoolers on account of its fewer cells with probabilities of less than 0.03 and good classification quality.

After the identification of the optimal latent transition model, the motor development patterns of the subgroup members were evaluated using the mean scores for each dimension. As shown in Table 3, the number of latent profiles in each transition model was identical to the former case in which the latent profiles were separately analyzed at each time point, although the motor development patterns were slightly different.

According to the mean scores for each of the dimensions, for the three-year-old preschoolers, the first, second, third, and fourth latent profiles were classified as subgroups named “low-to-moderate gross and low fine motor development,” “moderate-to-high gross and low fine motor development,” “high gross and moderate-to-high fine motor development,” and “moderate gross and moderate-to-high fine motor development,” respectively. The numbers of preschoolers in the four subgroups were 383 (20.33%), 812 (43.10%), 455 (24.15%), and 234 (12.42%), respectively.

For the four-year-old preschoolers, the first, second, and third latent profiles were classified as subgroups named “low-to-moderate gross and moderate fine motor development,” “high gross and moderate-to-high fine motor development,” and “moderate gross and fine motor development,” respectively. The numbers of preschoolers in the four subgroups were 56 (2.97%), 1491 (79.14%), and 337 (17.89%), respectively.

The latent transitions of motor development in preschoolers are presented in Table 4. It was found that the 383 preschoolers who belonged to the “low-to-moderate gross and low fine motor development” subgroup at three years old had probabilities of 8% (31 preschoolers), 53% (203 preschoolers), and 39% (149 preschoolers) of moving into the “low-to-moderate gross and moderate fine motor development,” “high gross and moderate-to-high fine motor development,” and “moderate gross and fine motor development” subgroups, respectively, at four years old. These results demonstrate that after one year, there were few preschoolers with no progression in motor development, and their fine motor skills tended to develop from a low to a moderate level. For approximately half of the preschoolers, their gross motor skills improved to a high level, while their fine motor skills developed to a moderate-to-high level. Some of the preschoolers displayed little progression in terms of both gross and fine motor development.

There were 812 preschoolers who belonged to the “moderate-to-high gross and low fine motor development” subgroup at three years old, who had a probability of 1% (8 preschoolers) of moving into the “low-to-moderate gross and moderate fine motor development” subgroup, a probability of 86% (698 preschoolers) of moving into the “high gross and moderate-to-high fine motor development” subgroup, and a probability of 13% (106 preschoolers) of moving into the “moderate gross and moderate fine motor development” subgroup. These results demonstrate that after one year, the gross motor development of most of the preschoolers had regressed, whereas their fine motor development had progressed to a high level. On the contrary, the gross motor skills for a few preschoolers developed to a high level, while their fine motor skills improved dramatically from a low level to a high level.

In addition, it was found that the 455 preschoolers who belonged to the “high gross and moderate-to-high fine motor development” subgroup at three years old had a probability of 0% of moving into the “low-to-moderate gross and moderate fine motor development” and “high gross and moderate-to-high fine motor development” subgroups and a probability of 100% of moving into the “moderate gross and moderate fine motor development” subgroup. These results demonstrate that after one year, all of the preschoolers with high gross motor development and moderate-to-high fine motor development displayed no progression in terms of fine motor development. Conversely, there was no regression in either gross or fine motor development.

There were 234 preschoolers who belonged to the “moderate gross and moderate-to-high fine motor development” subgroup at three years old, who had probabilities of 6% (14 preschoolers), 60% (approximately 140 preschoolers), and 34% (80 preschoolers) of moving into the “low-to-moderate gross and moderate fine motor development,” “high gross and moderate-to-high fine motor development,” and “moderate gross and fine motor development” subgroups, respectively, at four years old. These results demonstrate that after one year, a few preschoolers not only showed no progression in terms of both gross and fine motor development but also displayed a general regression in both cases. For over half of the preschoolers, their gross motor skills improved to a high level, whereas their fine motor skills remained at the same level. Finally, some of the preschoolers showed the same level in terms of their gross motor development and a little regression in terms of their fine motor development.

## 4. Discussion and Implications

This study investigated latent transitions in the motor development patterns of preschoolers based on data derived from a large database and collected using a motor development scale developed for preschoolers. The results demonstrated good construct validity for the motor development scale. This scale considered the motor development of preschoolers in terms of two subscales for assessing gross motor development and fine motor development, each of which consisted of two dimensions, namely, “stability and movement” and “body coordination” for gross motor development and “grasp and hand operation” and “vision and movement coordination” for fine motor development.

Prior to the LTA, the LPA demonstrated that motor development in the preschoolers was not homogeneous but rather consisted of several heterogeneous subgroups at each of the two time points. These findings contradicted former findings that considered motor development in samples to be homogeneous [3,4,5,6,7,8,9], but were similar to others that found heterogeneity in the motor development of children [11]. The subgroup members displayed different levels of development in terms of both gross and fine motor skills. After synthetic evaluations, four and three latent profiles were identified for the three- and four-year-old preschoolers, respectively. The latent profiles at each time point were similar after conducting the LTA, although some differences in the motor development patterns were observed.

Nine latent transition patterns were found, namely, “low gross motor retention and fine motor progression” (1→1, i.e., latent profile 1 at t_1_ → latent profile 1 at t_2_), “general high motor progression” (1→2), “general low motor progression” (1→3), “low gross motor regression and fine motor progression” (2→1 and 2→3), “low gross motor regression and high fine motor progression” (2→2), “general motor retention” (3→2), “general low motor regression” (4→1), “gross motor progression and fine motor retention” (4→2), and “gross motor retention and low fine motor regression” (4→3).

The “low gross motor retention and fine motor progression” transition (i.e., the gross motor skills were maintained at the low-to-moderate level from t_1_ to t_2_, whereas the fine motor skills developed from a low level at t_1_ to a moderate level at t_2_) may suggest that some preschoolers were given little time to engage in outdoor physical activities to develop their gross motor skills to reach a higher level [22,23], whereas they were given relatively more time to engage in indoor learning activities to develop their fine motor skills [24]. The “general high motor progression” transition (i.e., the gross motor skills developed from the low-to-moderate level at t_1_ to a high level at t_2_, whereas the fine motor skills developed from a low level at t_1_ to the moderate-to-high level at t_2_) may suggest that some preschoolers were given a large amount of time to engage in both outdoor physical activities and indoor learning activities to develop both their gross and fine motor skills [23,24,25,26]. Similarly, the “general low motor progression” transition (i.e., the gross motor skills developed from the low-to-moderate level at t_1_ to a moderate level at t_2_, whereas the fine motor skills developed from a low level at t_1_ to a moderate level at t_2_) may suggest that some preschoolers were given little time to engage in both outdoor physical activities and indoor learning activities to develop both their gross and fine motor skills. The “low gross motor regression and fine motor progression” transition (i.e., the gross motor skills developed from the moderate-to-high level at t_1_ to the low-to-moderate/moderate level at t_2_, whereas the fine motor skills developed from a low level at t_1_ to a moderate level at t_2_) may suggest that some preschoolers were given little or no time to engage in outdoor physical activities [27,28] but some time to engage in indoor learning activities to develop their fine motor skills. The “low gross motor regression and high fine motor progression” (i.e., the gross motor skills developed from the moderate-to-high level at t_1_ to a high level at t_2_, whereas the fine motor skills developed from a low level at t_1_ to the moderate-to-high level at t_2_) transition may suggest that some preschoolers were given little or no time to engage in outdoor physical activities [29,30,31] but much time to engage in indoor learning activities to develop their fine motor skills [32]. The “general motor retention” transition (i.e., the gross and fine motor skills were maintained at a high level and the moderate-to-high level from t_1_ to t_2_, respectively) may suggest that many preschoolers were given roughly the same amount of time to engage in outdoor physical activities and indoor learning activities to develop both their gross and fine motor skills [23,33], but the difficulties or challenges of these activities may not have been sufficient for the preschoolers to develop both their gross and fine motor skills to high levels [23]. The “general low motor regression” transition (i.e., the gross motor skills declined from a moderate level at t_1_ to the low-to-moderate level at t_2_, whereas the fine motor skills also declined from the moderate-to-high level at t_1_ to a moderate level at t_2_) may suggest that some preschoolers were given insufficient time to engage in both outdoor physical activities and indoor learning activities to develop both their gross and fine motor skills [30,34], or it may imply that the difficulties or challenges of these activities were too low to elicit the interest or engagement of the preschoolers. The “low gross motor progression and fine motor retention” transition (i.e., the gross motor skills developed from a moderate level at t_1_ to a high level at t_2_, whereas the fine motor skills were maintained at the moderate-to-high level from t_1_ to t_2_) may suggest that some preschoolers were given some time to engage in outdoor physical activities to develop their gross motor skills and roughly the same amount of time daily or weekly to engage in indoor activities to develop their fine motor skills [24,35]. However, it may also imply that the difficulties or challenges of these activities were too low to elicit the interest or engagement of the preschoolers [23]. The “moderate gross motor retention and low fine motor regression” transition (i.e., the gross motor skills were maintained at a moderate level from t_1_ to t_2_, whereas the fine motor skills declined from the moderate-to-high level at t_1_ to a moderate level at t_2_) may suggest that some preschoolers were given considerable time to engage in outdoor physical activities to develop their gross motor skills but less time daily or weekly to engage in indoor activities to develop their fine motor skills [1,36,37,38,39,40,41].

This work was intended to serve as a preliminary study to probe the possible existence of heterogeneous subgroups during motor development in preschoolers. For future studies, a re-examination of the present findings by including and controlling several internal factors (e.g., genetics, epigenetics, medical conditions, and history) is needed, and it would be desirable to incorporate antecedent variables to investigate the situational effects (e.g., interventions) on these motor development transitions to clarify whether similar patterns could be combined. Similarly, it would be beneficial to incorporate outcome variables to investigate whether different motor development patterns may exert different effects on short-term and/or long-term consequences. For practitioners (e.g., preschool teachers), the findings obtained by the LTA may aid in understanding that preschoolers may have distinct needs in terms of different motor development patterns and levels. The LTA results may encourage practitioners to increase the time and/or challenge of indoor and/or outdoor activities for subgroups exhibiting motor development regression. For example, a “general motor regression” subgroup of preschoolers would benefit from more time for and a greater difficulty of both kinds of activities.

## 5. Conclusions

Preschoolers showed largely different patterns with different levels in their gross and fine motor development at different ages. These findings confirmed that motor development in preschooler populations is not homogeneous, and suggested that this heterogeneity should be considered in future studies to prevent researchers from drawing misleading conclusions. From age three to age four, a large proportion of the preschoolers exhibited improvements or retentions in both gross and fine motor skills, whereas some of the preschoolers were classified into subgroups displaying “gross motor retention and fine motor progression,” “gross motor progression and fine motor retention,” “gross motor retention and fine motor regression,” and “gross motor regression and fine motor progression.” Few preschoolers exhibited “general motor regression.” Future studies are encouraged to identify reasons for regression and retention below high motor development levels in order to effectively design practices to promote preschoolers’ motor development.

## Figures and Tables

**Table 1:** Mean scores for each of the motor development dimensions and the numbers and proportions of preschoolers in each latent profile at the two time points (*N* = 1884).

Dimensions and Items	Latent Profiles for Three-Year-Old Preschoolers (Number/Proportion of Preschoolers)			
	1 (275/14.60%)	2 (566/30.04%)	3 (576/30.57%)	4 (467/24.79%)
Gross motor development				
Stability and movement	1.69	3.86	2.85	3.91
Body coordination	1.75	2.36	2.18	3.21
Fine motor development				
Grasp and hand operation	1.71	1.85	1.87	2.58
Vision and movement coordination	1.84	1.85	2.09	3.26
	Latent profiles for four-year-old preschoolers (number/proportion of preschoolers)			
	1 (1494/79.30%)	2 (334/17.73%)	3 (56/2.97%)	
Gross motor development				
Stability and movement	3.94	2.89	1.77	
Body coordination	3.15	2.47	2.14	
Fine motor development				
Grasp and hand operation	2.99	2.61	2.42	
Vision and movement coordination	3.22	2.75	2.46	

**Table 2:** Goodness-of-fit indices for the latent transition models of motor development in preschoolers (*N* = 1884).

Model (Parameters)	AIC	BIC	ABIC
2-2 transition model (27)	34,680.76	34,830.37	34,744.59
2-3 transition model (33)	33,823.97	34,006.83	33,901.98
3-2 transition model (33)	34,218.13	34,400.99	34,296.15
3-3 transition model (40)	3338.27	33,559.92	33,432.84
3-4 transition model (47)	32,774.57	33,035.01	32,885.69
4-3 transition model (47)	33,247.93	33,508.36	33,359.04
4-4 transition model (55)	32,726.26	33,031.02	32,856.29
4-5 transition model (63)	31,887.12	32,236.21	32,036.06
5-4 transition model (63)	32,260.21	32,609.30	32,409.15
5-5 transition model (72)	31,533.88	31,932.85	31,704.10
5-6 transition model (81)	31,656.94	32,105.77	31,848.44
6-5 transition model (81)	31,979.38	32,428.21	32,170.87
6-6 transition model (91)	31,422.12	31,926.36	31,637.25
6-7 transition model (101)	29,726.87	30,286.53	29,965.65
7-6 transition model (101)	31,345.88	31,905.54	61,584.67
7-7 transition model (112)	29,576.44	30,197.05	29,841.23
7-8 transition model (123)	29,536.17	30,217.73	29,826.96
8-7 transition model (123)	29,506.88	30,188.45	29,797.68

Note: The numbers to the left and right of the hyphen denote the number of latent profiles at the first and second time points, respectively.

**Table 3:** Mean scores for each of the motor development dimensions and the numbers and proportions of preschoolers in each latent profile in the latent transition model (*N* = 1884).

Dimension	Latent Profiles for Three-Year-Old Preschoolers (Number/Proportion of Preschoolers)			
	1 (383/20.33%)	2 (812/43.10%)	3 (455/24.15%)	4 (234/12.42%)
Gross motor development				
Stability and movement	2.06	3.58	3.85	2.94
Body coordination	1.68	2.36	3.17	2.46
Fine motor development				
Grasp and hand operation	1.67	1.90	2.53	2.04
Vision and movement coordination	1.57	1.71	3.42	3.12
	Latent profiles for four-year-old preschoolers (number/proportion of preschoolers)			
	1 (56/2.97%)	2 (1491/79.14%)	3 (337/17.89%)	
Gross motor development				
Stability and movement	1.77	3.94	2.90	
Body coordination	2.14	3.15	2.46	
Fine motor development				
Grasp and hand operation	2.42	2.99	2.61	
Vision and movement coordination	2.45	3.22	2.74	

**Table 4:** Transition probabilities in the latent transition models.

Latent Profile at t_1_ (Number/Proportion of Preschoolers)	Latent Profile at t_2_ (Gross, Fine Motor Development) (Number of Preschoolers)		
	Low-to-Moderate, Moderate	High, Moderate-to-High	Moderate, Moderate
Low-to-moderate, low (383/20.33%)	0.08 (31)	0.53 (203)	0.39 (149)
Moderate-to-high, low (812/43.10%)	0.01 (8)	0.86 (698)	0.13 (106)
High, moderate-to-high (455/24.15%)	0.00 (0)	1.00 (455)	0.00 (0)
Moderate, moderate-to-high (234/12.42%)	0.06 (14)	0.60 (140)	0.34 (80)

## Data Availability

This article used publicly available data with no protected health Information, and the data can be derived from https://srda.sinica.edu.tw/datasearch_detail.php?id=2953 (accessed on 15 March 2023).
